# Yeast Use Dual Gain Controls to Amplify Protein Processing

**DOI:** 10.1371/journal.pbio.0020269

**Published:** 2004-08-17

Machinery within the endoplasmic reticulum (ER) of eukaryotic cells modifies, folds, and assembles proteins as needed to suit their functions at or past the cell membrane. When this system is hampered or overtaxed, a buildup of unfolded or misfolded proteins within the ER triggers the “unfolded protein response,” which alerts the nucleus to boost production of protein-processing machinery that helps proteins fold. This system for adjusting manufacturing capacity is similar in organisms from yeast to human. If the unfolded protein response cannot be turned on when needed, cells die. Prior study suggested that, in yeast cells, the response to unfolded protein buildup is binary: either off or on. In this month's *PLoS Biology*, biochemist Peter Walter and his colleagues from the University of California at San Francisco demonstrate two new signaling mechanisms that appear to give the yeast unfolded protein response the means for amplitude adjustment.

In yeast, a transcription regulator called Hac1p activates the genes required for the unfolded protein response. A cytoplasmic pool of *HAC1* messenger RNA waits in readiness for ER emergencies, each molecule locked against translation into protein by intronic RNA sequences that interrupt mRNA translation. Accumulation of unfolded or misfolded proteins in the ER releases this translational block, triggering production of Hac1p and activating the unfolded protein response. Previously, this binary *HAC1* signal was the only known regulator of the unfolded protein response in yeast. In two complementary papers, Walter and colleagues now present evidence that the repertoire includes new factors and regulators that amplify the unfolded protein response under conditions of ER stress.[Fig pbio-0020269-g001]

**Figure pbio-0020269-g001:**
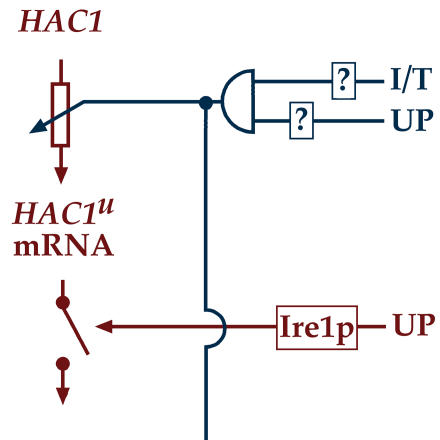
Wiring the unfolded protein response

Leber et al. stressed yeast cells by exposing them to substances that cause protein misfolding or buildup in the ER. In response, the cells ratcheted up transcription levels of *HAC1* severalfold. Primed with high levels of *HAC1* mRNA, the cells were ready to produce a bumper crop of Hac1p and to induce a supercharged unfolded protein response. In the accompanying paper by Patil et al., the authors show that Hac1p is not working alone. A second regulator of transcription called Gcn4p is required to activate most of the genes associated with the unfolded protein response. The regulatory elements of these genes now appear far more diverse than previously appreciated. The authors propose that cells adjust the levels of Hac1p and Gcn4p to drive a continuum of transcriptional programs equipped to deal with incoming challenges. Together, these two papers demonstrate that the control of the unfolded protein response is far from a simple on/off mechanism, but exhibits complex fine-tuning through a network of signaling pathways that interpret and respond to the cell's needs.[Fig pbio-0020269-g002]

**Figure pbio-0020269-g002:**
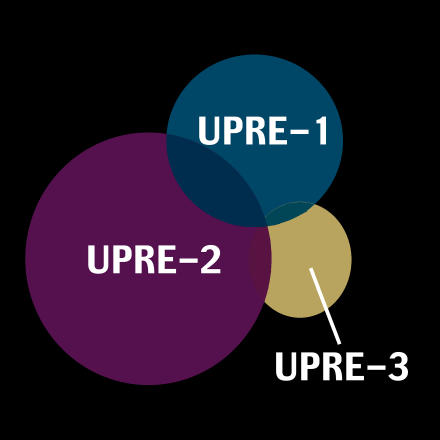
Control elements of UPR target genes

